# LONGITUDINAL ASSOCIATIONS OF DEPRESSIVE SYMPTOM AND SUBJECTIVE MEMORY FACTORS WITH OBJECTIVE MEMORY PERFORMANCE

**DOI:** 10.1093/geroni/igz038.2406

**Published:** 2019-11-08

**Authors:** Wonjeong Haavisto, Julie Blaskewicz Boron, Sherry Willis, Warner Schaie

**Affiliations:** 1 University of Nebraska, Omaha, Nebraska, United States; 2 University of Nebraska at Omaha, Omaha, Nebraska, United States; 3 University of Washington, Seattle, Washington, United States

## Abstract

Prior research demonstrated a history of depressive symptoms predicted oncoming memory deficits, and that self-evaluation of memory was associated with forthcoming memory difficulties. However, prior work lacks consistent consideration of multifaceted depressive symptoms in regards to longitudinal associations with objective memory (OM). Structural models were examined to determine how latent factors of depressive symptoms (via the CES-D) and SM factors predicted memory deficits at later time points when taking into account baseline OM performance [n=270; RMSEA=.034; CFI=.974; TLI=.965] in the Seattle Longitudinal Study (mean Age=70.33; SD=7.29; mean Education=15.30; SD=2.72; 61.9% female). The somatic complaints CES-D factor showed a significant longitudinal association with OM performance after seven years (β = -.25, p < .05), while none of CES-D factors showed cross-sectional associations with the baseline OM. The general frequency of forgetting SM factor was positively associated with OM performance at baseline (β = .26, p < .001), suggesting that those performing better at recalling words reported fewer memory problems. None of SM factors showed longitudinal associations with OM measured seven years later, indicating that self-evaluation of memory had no impact on future memory deficits. Overall findings suggested that a key CES-D factor, somatic complaints, was detected and that people endorsing more somatic issues experienced greater memory decline over a seven year period. Thus, extending prior work, the current study suggests that although both subjective memory and depressive symptom factors showed concurrent associations, only a specific factor of depressive symptoms, somatic complaints, was influential in regards to predicting later memory performance

